# Rayleigh step-selection functions and connections to continuous-time mechanistic movement models

**DOI:** 10.1186/s40462-023-00442-w

**Published:** 2024-02-08

**Authors:** Joseph M. Eisaguirre, Perry J. Williams, Mevin B. Hooten

**Affiliations:** 1 U.S. Geological Survey, Alaska Science Center, Anchorage, AK USA; 2 Department of Natural Resources & Environmental Science, University of Nevada, Reno, NV USA; 3 Department of Statistics and Data Sciences, The University of Texas at Austin, Austin, TX USA

**Keywords:** Change of variables, Continuous-time, Ecological diffusion, First principles, Fokker-Planck, Habitat selection, Partial differential equation, Step-selection function, Resource selection function, Space use

## Abstract

**Background:**

The process known as ecological diffusion emerges from a first principles view of animal movement, but ecological diffusion and other partial differential equation models can be difficult to fit to data. Step-selection functions (SSFs), on the other hand, have emerged as powerful practical tools for ecologists studying the movement and habitat selection of animals.

**Methods:**

SSFs typically involve comparing resources between a set of used and available points at each step in a sequence of observed positions. We use change of variables to show that ecological diffusion implies certain distributions for available steps that are more flexible than others commonly used. We then demonstrate advantages of these distributions with SSF models fit to data collected for a mountain lion in Colorado, USA.

**Results:**

We show that connections between ecological diffusion and SSFs imply a Rayleigh step-length distribution and uniform turning angle distribution, which can accommodate data collected at irregular time intervals. The results of fitting an SSF model with these distributions compared to a set of commonly used distributions revealed how precision and inference can vary between the two approaches.

**Conclusions:**

Our new continuous-time step-length distribution can be integrated into various forms of SSFs, making them applicable to data sets with irregular time intervals between successive animal locations.

**Supplementary Information:**

The online version contains supplementary material available at 10.1186/s40462-023-00442-w.

## Background

The ecological diffusion equation (EDE) is a mathematical description of the probability of presence for animals in space and time, which can be obtained by starting with a simple set of first principles defining how an individual animal can move across a landscape [17]. Ultimately, a stochastic differential equation for individual trajectories gives rise to the Fokker-Planck equation, and taking the limit with respect to time and space results in the partial differential equation (PDE) known as the EDE [35]. A suite of contemporary statistical modeling approaches have relied on the EDE to characterize the growth and spread of animal populations [7, 14, 16, 21, 36]. In particular, Hooten et al. [18] recently introduced a mechanistic individual-level modeling framework based on ecological diffusion and aligned it with step-selection functions (SSFs, or step-selection analyses; [11, 34]). SSFs are commonly applied by practitioners to study the movement and resource selection of animals tagged with modern telemetry devices [8].

Fortin et al. [11] coined the term and are often credited with implementing the first SSF in an application studying the resource selection of elk (*Cervus canadensis*). The motivation was to overcome challenges associated with defining what is ‘available’ to an animal when implementing conventional resource selection functions (RSFs); an SSF does so by letting properties of the animal’s movement define the availability function [10, 34], while availability is often somewhat arbitrary for RSFs [22]. The method implemented by Fortin et al. [11] was a first practical step; however, SSFs have since been studied rigorously, including establishing connections to mechanistic home range models [25] and similar differential equation models [26, 30], space-time point process (STPP) models [19], and other popular contemporary movement modeling approaches, such as certain forms of random walks (e.g., [3]). SSFs have also seen development in terms of estimation procedures both at the individual-level [2, 32] and population-level [24].

As with RSFs, SSFs typically assume an exponential selection function. This is despite limitations—primarily that inference is restricted to ‘relative’ selection strength instead of absolute probability of use, as can be inferred with a logistic selection function, for example [1, 20]. However, the exponential form permits estimation using a suite of readily available software commonly used in conditional logistic regression (recently discussed by Muff et al. [24]). While such convenience makes SSFs approachable to practitioners, ecological diffusion implies a different form of selection function that directly relates resource and habitat selection to residence time [18]. Although the EDE SSF presented by Hooten et al. [18] can still be implemented in a conditional regression framework, the implied link function is not standard and thus requires custom algorithms to fit the model to data.

Irrespective of the form of selection function, many regression-based approaches for fitting SSF models to data involve comparing the habitat or resources at ‘used’ steps to those at ‘available’ (or ‘control’) steps [2, 10, 34]. This procedure overcomes the need to evaluate an integral in the SSF likelihood that is usually analytically intractable. Generating available steps is typically done with distributions of step lengths (or distances between successive observed locations of the animal) and turning angles (or the angular deviations between steps; [34]). There have been various suggestions for how to choose these distributions, including empirical and parametric forms. Parametric choices for step lengths include the log-normal, gamma, and exponential, and uniform and von Mises are common for turning angles [2, 10, 34]. Many of these recommendations have resulted from rigorous simulation studies [2, 10].

In contrast to common formulations based on polar coordinates (i.e., step lengths and turning angles), a homogenized version of the EDE SSF implies a multivariate normal kernel on Cartesian coordinates for the availability distribution [18]. While similarly useful for sampling available steps in an SSF procedure, it has been used only infrequently (e.g., [5]), and polar coordinate formulations have been the focus of SSF developments and in application. In large part limited by the polar coordinate formulation, conventional SSFs are typically restricted to discrete-time frameworks and thus require regular sampling intervals in telemetry data [2, 10, 34].

In what follows, we show that first principles of animal movement and ecological diffusion imply certain parametric forms for the step-length and turning angle distributions that are not commonly used nor currently recommended in the SSF literature. Doing so reveals a continuous-time, polar coordinate formulation of the movement kernel, which naturally accommodates data sets with irregular sampling intervals and/or missing data, while still maintaining the form familiar to practitioners. Additionally, we show how the EDE SSF presented by Hooten et al. [18] can be implemented by generating available steps using step-length and turning angle distributions, which further aligns it with other familiar SSF estimation procedures. To illustrate advantages of using the continuous-time EDE availability distributions, we also apply a traditional exponential SSF to GPS data collected for a mountain lion in Colorado with irregular sampling intervals using both the EDE distributions and commonly recommended distributions. Finally, we discuss how the EDE distributions can be incorporated into other types of SSFs, including the popular integrated step-selection analysis (iSSA; [2, 32]).

## Methods

SSFs, which approximate STPP models, can be expressed using a weighted distribution form, such that

1 $$\begin{aligned}{}[\textbf{s}(t_i)|\textbf{s}(t_{i-1}),\varvec{\beta }] \equiv \frac{g(\textbf{w}(\textbf{s}(t_i)),\varvec{\beta })f_i(\textbf{s}(t_i)|\textbf{s}(t_{i-1}))}{\int _{\mathcal {S}}g(\textbf{w}(\textbf{s}),\varvec{\beta })f_i(\textbf{s}|\textbf{s}(t_{i-1}))d\textbf{s}}, \end{aligned}$$

where the bracket notation [*a*|*b*] represents a probability distribution of *a* given *b* [13], $\textbf{s}(t_i)$ is a Cartesian coordinate vector of the animal’s location at time $t_i$ for $i=1,\dots ,n$ steps, $f_i(\textbf{s}(t_i)|\textbf{s}(t_{i-1}))$ is a movement (or availability) kernel, and $g(\textbf{w}(\textbf{s}(t_i)),\varvec{\beta })$ weights the movement kernel based on the habitat or resources available [17, 18].

The ecological diffusion equation (EDE; i.e., Fokker-Plank PDE) can be written [35]:

2 $$\begin{aligned} \frac{\partial }{\partial t} p(\textbf{s},t) = \left( \frac{\partial ^2}{\partial s^2_1} + \frac{\partial ^2}{\partial s^2_2}\right) \delta (\textbf{s}) p(\textbf{s},t), \end{aligned}$$

where $p(\textbf{s},t)$ is the probability of presence over the continuous temporal domain $t\in (0,\infty )$ and the continuous spatial domain $\textbf{s}\equiv (s_1,s_2)' \in \mathcal {S}$, and $\delta (\textbf{s})$ is motility, which is inversely proportional to residence time. [18] showed that homogenization, a mathematical technique commonly used to reduce the computational burden of statistical PDE models [6, 12, 14, 16, 21, 36], yields the fundamental solution to (2):

3 $$\begin{aligned}{}[\textbf{s}(t_i)|\textbf{s}(t_{i-1}),\varvec{\beta }] \propto \frac{1}{\delta (\textbf{s}(t_i))\Delta t_i} \text {exp}\left( -\frac{1}{2}(\textbf{s}(t_i)-\textbf{s}(t_{i-1}))'(2\bar{\delta }(t_i)\Delta t_i \textbf{I})^{-1}(\textbf{s}(t_i)-\textbf{s}(t_{i-1}))\right) , \end{aligned}$$

where $\bar{\delta }$ is the homogenized motility coefficient and $\textbf{I}$ is the identity matrix. Further, $\bar{\delta }(t_i)$ can be pre-estimated with a temporal moving average of the telemetry data, such that

4 $$\begin{aligned} \bar{\delta }(t_i) \approx \sum _{t_j \sim t_i} \frac{(\textbf{s}(t_j)-\textbf{s}(t_{j-1}))'(\textbf{s}(t_j)-\textbf{s}(t_{j-1}))}{4n_i\Delta t_j}, \end{aligned}$$

where $t_j\sim t_i$ is the set of times $t_j$ that are temporally close to $t_i$, $\Delta t_j$ is the time interval between $t_{j-1}$ and $t_j$, and $n_i$ is the size of that set [18]. Choosing the temporal span of $t_j$ will be study dependent, but generally a larger span will result in smoother changes to the movement kernel through time.

Equation (3) is the product of a multivariate normal availability kernel and a non-traditional selection function that can be interpreted directly in terms of residence time (i.e., the inverse of motility; [18]). Specifically,

5 $$\begin{aligned} g(\textbf{w}(\textbf{s}(t_i)),\varvec{\beta })=\frac{1}{\delta (\textbf{s}(t_i))\Delta t_i}, \end{aligned}$$

where, $\delta (\textbf{s}(t_i))=\frac{\Delta \textbf{s}^2}{4\Delta t_i}\text {logit}^{-1}(\textbf{w}'(\textbf{s}_i)\varvec{\beta })$, $\Delta s^2$ is the spatial grain, and

6 $$\begin{aligned} f_i(\textbf{s}(t_i)|\textbf{s}(t_{i-1}))\propto \text {exp}\left( -\frac{1}{2}(\textbf{s}(t_i)-\textbf{s}(t_{i-1}))'(2\bar{\delta }(t_i)\Delta t_i \textbf{I})^{-1}(\textbf{s}(t_i)-\textbf{s}(t_{i-1}))\right) . \end{aligned}$$

Based on (6), it is clear that estimating $\varvec{\beta }$ in this EDE SSF with the conventional conditional use-availability scheme requires generating available steps from a multivariate normal distribution with mean $\textbf{s}(t_{i-1})$ and variance-covariance matrix $2\bar{\delta }(t_i)\Delta t_i \textbf{I}$ [18]. However, this contrasts with most SSF implementations, which use an availability distribution parameterized in terms of polar coordinates—combinations of step-length and turning angle distributions.

### Step lengths and turning angles implied by ecological diffusion

Deriving step-length and turning angle distributions implied by the multivariate normal availability distribution for the EDE SSF involves a change of variables [28, 29]. We first let $(\textbf{s}(t_i)-\textbf{s}(t_{i-1}))=\textbf{h}(l_i,\theta _i)=(l_i\text {cos}(\theta _i), l_i\text {sin}(\theta _i))'$, where $l_i=||\textbf{s}(t_i)-\textbf{s}(t_{i-1})||$ and $\theta _i$ is the turning angle (i.e., difference in the animal’s headings between moves from $\textbf{s}(t_{i-2})$ to $\textbf{s}(t_{i-1})$ and $\textbf{s}(t_{i-1})$ to $\textbf{s}(t_{i})$); this implies $\textbf{h}(l_i,\theta _i)$ maps step lengths and turning angles to Cartesian coordinates. We then seek the joint density $[l_i,\theta _i]$. The change of variables is

7 $$\begin{aligned}{}[l_i,\theta _i]=[\textbf{h}(l_i,\theta _i)]|\textbf{J}(\textbf{h}(l_i,\theta _i))|, \end{aligned}$$

where $\textbf{J}$ is the Jacobian. The Jacobian in this case is defined as

8 $$\begin{aligned} \textbf{J}(\textbf{h}(l_i,\theta _i))\equiv \begin{pmatrix} \frac{\partial }{\partial l_i} s_1(t_i) &{} \frac{\partial }{\partial \theta _i}s_1(t_i) \\[1ex] \frac{\partial }{\partial l_i} s_2(t_i) &{} \frac{\partial }{\partial \theta _i}s_2(t_i) \end{pmatrix}, \end{aligned}$$

and its determinant is

9 $$\begin{aligned} |\textbf{J}(\textbf{h}(l_i,\theta _i))|=l_i\text {cos}^2(\theta _i)+l_i\text {sin}^2(\theta _i)=l_i. \end{aligned}$$

Thus, $[l_i,\theta _i]=[\textbf{h}(l_i,\theta _i)]l_i$. Therefore, we arrive at $[l_i,\theta _i]$ by substituting $\textbf{h}(l_i,\theta _i)$ into (6) and multiplying by $l_i$, which results in

10 $$\begin{aligned}{}[l_i,\theta _i]&=\frac{l_i}{2\bar{\delta }(t_i)\Delta t_i}\text {exp}\left( \frac{-l_i^2}{4\bar{\delta }(t_i)\Delta t_i}\right) , \end{aligned}$$

implying $l_i \sim \text {Rayleigh}(\sigma ^2)$, where $\sigma ^2=2\bar{\delta }(t_i)\Delta t_i$, and $\theta _i \sim \text {Unif}(0,2\pi )$. Therefore, $[l_i,\theta _i]$ can be substituted for $f_i$ in (1), and sampling available steps using these distributions is equivalent to sampling from the multivariate normal density in (6). A key difference between these and other step-length and turning angle distributions suggested for SSFs is that (10) depends on $\Delta t_i$, implying that this formulation (and the one presented by Hooten et al. [18]) is continuous-time and may be applied to data collected with unequal time intervals between fixes.

### Continuous-time movement SSF: case study with mountain lion data

To demonstrate the advantages of using the continuous time EDE availability distributions, as well as their potential for increasing the flexibility of a wide range of SSF models, we applied an exponential SSF to GPS data collected for a mountain lion in Colorado using both the EDE distributions and a commonly recommended set of distributions. These data were previously analyzed in case studies by Hooten et al. [17, 18]. The data consist of $n=150$ locations spanning 2.5 weeks. The time interval ($\Delta t_i$) between locations varied primarily between 3 hr (83 steps) and 4 hr (60 steps), but some intervals were up to 8 hr. Following Hooten et al. [18], we used aspect, elevation, and slope as covariates in the SSF.

We fit the exponential SSF, where $g(\textbf{w}(\textbf{s}(t_i)),\varvec{\beta }) \equiv \text {exp}(\textbf{w}(\textbf{s}(t_i))'\varvec{\beta })$, to the full data set using the Rayleigh step-length distribution and uniform turning angle distribution presented above. We first computed $\bar{\delta }(t_i)$ following Hooten et al. [18], and then generated a set of 100 available locations per used location. We also increased this to 500, but the results did not change appreciably. The computed values of $\bar{\delta }(t_i)$ are not necessarily intended for making ecological inference but rather are used to facilitate estimation, similar to conventional SSF fitting procedures [34]. Finally, we used the function ‘clogit’ from the survival package in R to implement a conditional logistic regression and estimate the selection coefficients [27, 33]. Confidence intervals (CIs) for the selection coefficients were obtained using the function ‘confint’.

We also fit the SSF using a conventional set of distributions. However, this required a data set with a regular sampling interval [34], so we fit this SSF to only the steps where $\Delta t_i=3$ hr. Following Avgar et al. [2] and Signer et al. [32], we chose the gamma distribution for step lengths. Investigating the empirical turning angle distribution suggested little to no apparent directional persistence, so we used a uniform turning angle distribution, as above. We fit the gamma distribution to the data with standard maximum likelihood methods in R using ‘optim’ and estimated the selection coefficients with ‘clogit’.

## Results

**Fig. 1:**
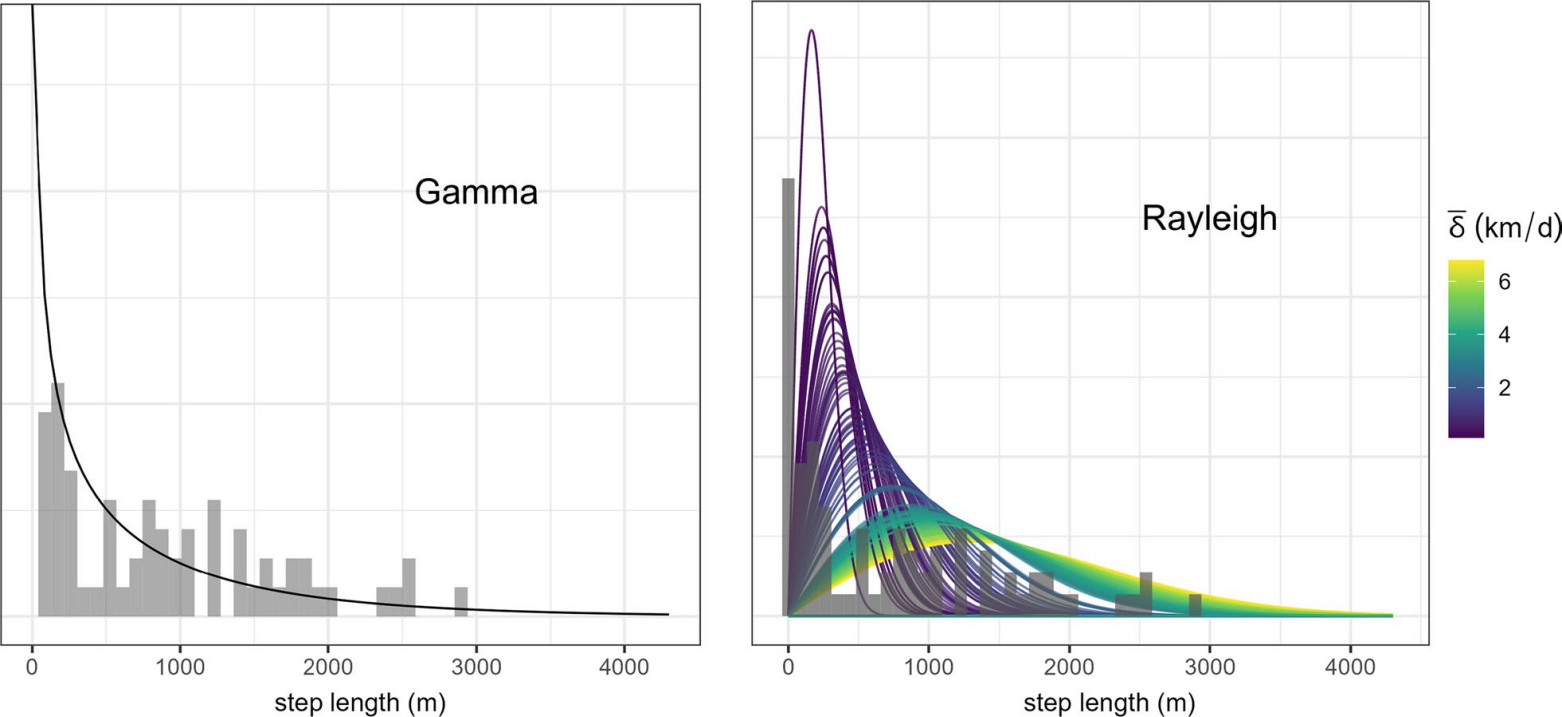
Fitted (curves) and empirical (bars) step-length distributions for the mountain lion tracked in Colorado. Each curve for the Rayleigh corresponds to an estimated value of the homogenized motility coefficient $\bar{\delta }_i$ from the ecological diffusion model and is scaled to $\Delta t_i=3$ hr

**Fig. 2:**
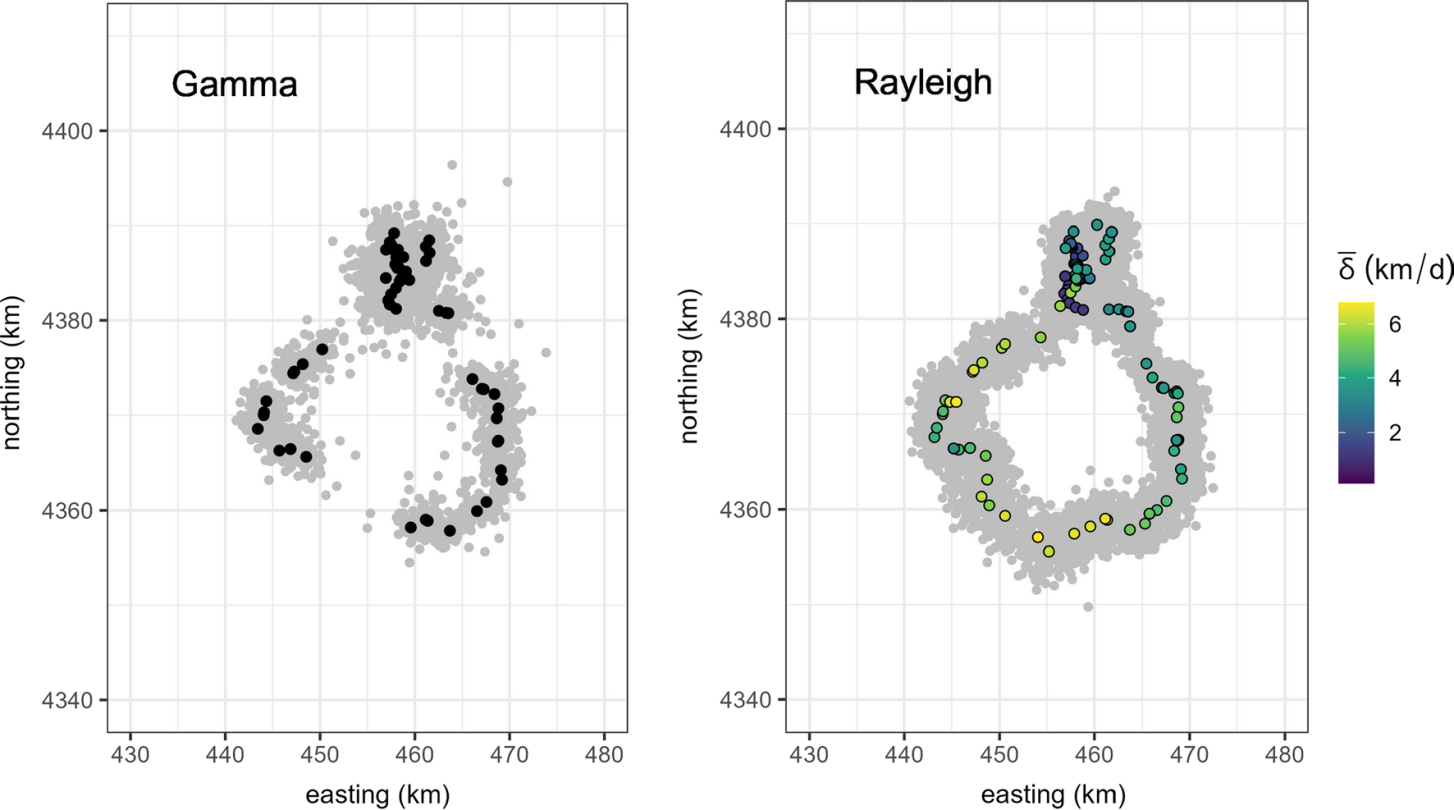
Used (black/hue) and available (gray) points for the mountain lion tracked in Colorado generated with gamma and Rayleigh step-length distributions. 100 available steps were generated for each used step. Hue for the Rayleigh distribution corresponds to homogenized motility from the ecological diffusion model, where higher values correspond to a faster movement rate (and lower residence time)

The empirical and fitted gamma and Rayleigh step-length distributions are shown in Fig. 1. Availability sets generated from the distributions varied, in part driven by the restricted data set required to meet the discrete-time assumptions of the conventional (gamma) step-length distribution (Fig. 2).

**Fig. 3:**
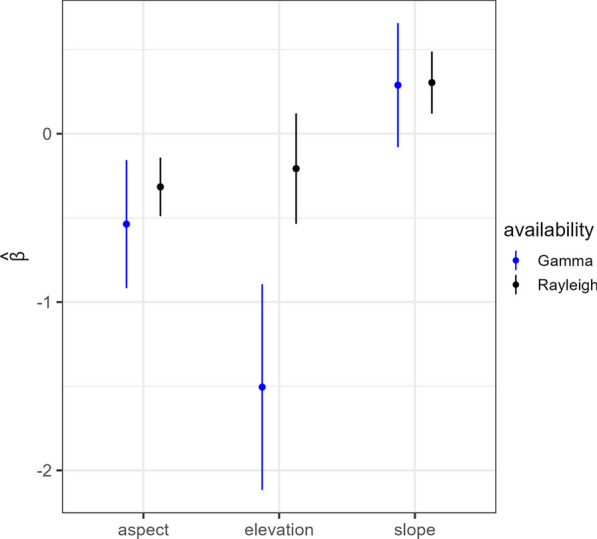
Estimated selection coefficients and 90% confidence intervals from the exponential SSF fit to data from a mountain lion tracked in Colorado using gamma and Rayleigh step-length distributions

Using the Rayleigh step-length distribution and full data set yielded more precise estimates of $\varvec{\beta }$ (Fig. 3). Even restricting the Rayleigh SSF to the sub-sampled data set still yielded slight gains in precision and was a better fit than the gamma SSF (Additional file 1: Fig. S1). Although, we note that in all cases estimated uncertainty could be biased low due to not jointly estimating the movement and resource selection parameters.

We also found that ecological inference could be affected depending on the distributions and requisite data sets used. In particular, a practitioner using the gamma distribution (and restricted data set) would infer elevation having a strong effect on mountain lion space use and slope having no significant effect (i.e., because the CI overlaps zero; Fig. 3). In contrast, a practitioner using the Rayleigh distribution paired with the full data set might infer aspect and slope, but not elevation, having significant effects (Fig. 3).

## Discussion

While the combinations of step-length and turning angle distributions—comprising the availability distributions in SSFs—currently recommended in the literature have been shown to yield unbiased inference about animal movement and resource selection (e.g., [2, 10]), we have shown that a first principles view of animal movement and ecological diffusion yield a new step-length distribution not commonly used in SSFs. We found that this new form also naturally accommodates data sets with varying sampling intervals, which overcomes a significant limitation of most current SSF formulations [15].

Our application to the mountain lion data comparing the continuous-time Rayleigh formulation and the conventional gamma formulation revealed notable differences. In particular, the Rayleigh SSF yielded greater precision associated with the selection coefficients. However, inferred effects of the covariates were also influenced by the step-length distribution and the restricted data set required for the gamma formulation (Figs. 2, 3, and Additional file 1: Fig. S1). Furthermore, Hooten et al. [17] applied an SSF using a conditional circular availability distribution to the same mountain lion data. Although they used a Bayesian Poisson regression, their results were similar to ours with the Rayleigh distribution, and it is possible the Rayleigh implementation yielded better precision (Fig. 3).

Although the Rayleigh step-length and uniform turning angle distributions we derived are equivalent to the multivariate normal availability distribution presented by Hooten et al. [18], the exponential weighting (or selection) function we implemented is different than what is implied by the EDE, which affected inference. Specifically, the full EDE SSF represented by (5) and (6) yields inference about movement probabilities and thus residence time directly, whereas the exponential weighting function is limited to relative selection strength. It is therefore not surprising that our coefficient estimates implied effects in the opposite direction compared to EDE SSF estimates from Hooten et al. [18], who found that the mountain lion exhibited higher residence time (lower motility) in areas at lower elevation, with steeper slopes, and with less exposed aspects. Using the ‘selection’ terminology, we might infer from our results here that the mountain lion ‘selected for’ steeper slopes and ‘selected against’ exposed aspects and higher elevations, which is analogous to, but less mechanistic, than the inference from the EDE SSF.

Although SSFs can be viewed as approximations of STPP models, two classes have emerged: one where the movement (or availability) kernel parameters are estimated *a priori* (e.g., [11]), and another where the movement and resource selection parameters are estimated jointly (e.g., [2]). So-called integrated step-selection analysis (iSSA; [2]) has become a popular tool for ecologists studying the movement and resource selection of animals. This is partly due to how the method “integrates” the movement parameters (i.e., parameters of the step-length and turning angle distributions) into the conditional logistic regression, allowing for richer forms of weighting functions (e.g., interactions between habitat covariates and movement) and recovering the theoretical selection-independent movement kernel [2]. Fieberg et al. [8]  discuss some of the advantages to this approach. Software developments, such as the R package amt [32], have also made the method accessible.

Arriving at the iSSA likelihood requires assuming an underlying step-length distribution belonging to the exponential family, such as the exponential and gamma distributions. However, the Rayleigh distribution also belongs to the exponential family. It can thus be shown that the Rayleigh distribution can be used in iSSA, which expands the utility of iSSA to data sets with varying sampling intervals. Although we did not implement an iSSA for our case study, it could be coupled with our approach by following the derivation of Avgar et al. [2] [8]. One could then use a simulation procedure to estimate the underlying utilization distribution [2, 31, 32] but accounting for potential irregular sampling intervals [9, 15]. Unlike the selection function derived by Hooten et al. [18], the exponential selection function we used to demonstrate the continuous-time Rayleigh step legnth distribution does not imply a fully continuous time SSF. Therefore it does not alleviate the issue of “scale-dependence” in SSFs, but rather provides a means to account for irregular data [15].

While the Rayleigh step-length distribution has the potential to benefit various forms of SSFs, in large part due to its continuous-time properties, other forms of step-length and turning angle distributions have roots in PDEs as well. For example, it can be shown that modeling step lengths divided by $\sqrt{\Delta t_i}$ with a Rayleigh distribution and uniform turning angles would imply plain diffusion, which, although simpler than estimating $\bar{\delta }(t_i)$, does not account for potential spatial variation in motility like the EDE availability distributions. Further, Moorcroft and Lewis [23] showed that exponentially distributed step lengths and turning angles distributed von Mises imply a certain form of advection–diffusion PDE. The diffusion component of that PDE differs from the EDE in that the *diffusion coefficient* lies outside of the partial derivatives (or differential operator), whereas *motility* lies inside both derivatives in the EDE, which affects how the environment can drive movement and probability of presence [12, 18, 35]. Like the EDE, advection–diffusion PDEs are appearing more in recent movement ecology literature as connections to SSFs (and their convenient estimation methods) are recognized (e.g., [26]). Advection-diffusion PDEs have the advantage of accounting for potential directional persistence or bias in movement, and step length and turning angle distributions for any form of PDE can be derived using the change-of-variables approach we used here for the EDE.

## Conclusions

A first principles view of animal movement establishes links between mechanistic models and SSFs, and studying those connections for implied forms of SSFs will allow for richer and more rigorous inference. In some cases, those forms may already appear in the literature (e.g., [23]), but in other cases it may be worthwhile to utilize mathematical techniques, such as homogenization [18] and change of variables, to identify new ways to improve inference about animal movement ecology.

### Supplementary Information

**Additional file 1.** Supplementary figure.

## Data Availability

Data can be found associated with Hooten et al. [17, 18]. Code is available from Eisaguirre [4].
