# Supplementary material for: Rayleigh step-selection functions and connections to continuous-time mechanistic movement models
Source: Mov Ecol. 2024 Feb 8;12:14. doi: 10.1186/s40462-023-00442-w (PMC10854073; doi:10.1186/s40462-023-00442-w)
Supplement: Supplementary file 1 — Additional file 1. Supplementary figure. [file 40462_2023_442_MOESM1_ESM.pdf]

Appendix 1: supplementary figure

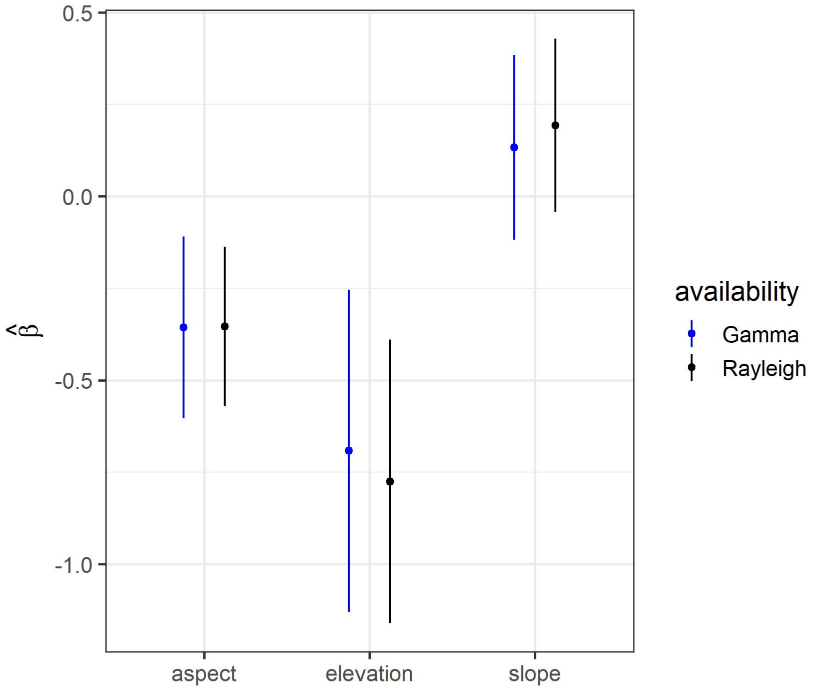

Figure S1: Estimated selection coefficients and 90% confidence intervals from the exponential SSF fit to data from a mountain lion tracked in Colorado using gamma and Rayleigh step-length distributions. Both were fit to the data subset to a fixed 3-hr time interval. In addition to the slight increase in precision, AIC for the Rayleigh SSF was 752 and AIC for the gamma SSF was 758, suggesting the Rayleigh SSF was a better fit.
